# Latent profile analysis of rural foreign language teacher resilience: its associations with emotional and professional adaptation

**DOI:** 10.3389/fpsyg.2026.1906687

**Published:** 2026-08-04

**Authors:** Ting Huang

**Affiliations:** Department of Foreign Languages, Fujian Institute of Education, Fuzhou, China

**Keywords:** emotion regulation, emotional labour, latent profile analysis, rural foreign language teachers, teacher professional identity, teacher resilience, teacher subjective wellbeing

## Abstract

Teacher resilience, emotional adaptation, and professional adaptation are central to how foreign language teachers satisfy the demands of their work, but they have rarely been examined within a single framework. Using a cross-sectional quantitative survey design, the present study investigated how multidimensional resilience is configured among rural foreign language teachers in China's basic education and how resilience configurations relate to teachers' emotional and professional adaptation. Data were collected from 262 in-service junior secondary English teachers (81.7% female; mean teaching experience = 18.75 years) who work predominantly in county- and township-level schools in rural Fujian Province, China. Confirmatory factor analysis supported the 16-dimension measurement model, and latent profile analysis identified four resilience profiles: Frail, Balanced, Hardcore, and Outreaching. Profile membership was systematically associated with eleven of the twelve adaptation outcomes, following a resilience gradient in which Hardcore teachers reported the most adaptive functioning and Frail teachers the least; surface acting was the only outcome unrelated to profile membership. The findings support the view of teacher resilience as a foundational, configurally organised adaptive resource. This research thus suggests that support for rural foreign language teachers should build personal competences and meaning alongside relational support.

## 1 Introduction

In foreign language education, teacher resilience assumes particular importance because language teaching involves a distinctive combination of target-language management, affective regulation, corrective-feedback work, and intercultural mediation (Kohler, 2015; Liddicoat and Scarino, 2013; Tao et al., 2024). Foreign language teachers are expected to sustain meaningful target-language interaction, regulate learners' anxiety and motivation, provide continuous corrective feedback, and navigate culturally mediated classroom relationships, all of which intensify the emotional and professional demands of teaching (Turnbull and Arnett, 2002). Within this domain, teacher resilience has emerged as an increasingly important construct, given its close associations with teachers' psychological adaptation, such as emotion regulation and emotional labour, and their professional adaptation, as reflected in subjective wellbeing and professional identity. These dimensions are not isolated from one another. Rather, they represent interconnected aspects of how teachers respond to the demands of teaching, regulate their emotional experiences, sustain positive professional functioning, and maintain a stable sense of self within the profession. In this sense, teacher resilience may be understood as a foundational adaptive resource that supports both the emotional processes and the professional outcomes associated with effective teaching. These demands may be particularly consequential for teachers working in rural or county-level schools in China, where professional development opportunities, instructional resources, peer networks, and access to specialist support may be more limited than in urban schools. In such contexts, teacher resilience is not merely an individual psychological resource but also an adaptive capacity shaped by uneven contextual resources.

Notwithstanding the growing interest in teacher resilience, several gaps remain in the existing literature. First, although resilience has been linked to teachers' emotional and professional functioning, these relationships have often been examined in a piecemeal fashion, with separate studies focusing on isolated outcomes rather than a more integrated pattern of adaptation. Second, while teacher resilience is increasingly understood as a multidimensional construct, relatively little is known about whether different resilience dimensions combine in distinct ways within individual teachers. Third, this limitation is particularly salient in foreign language education, especially for rural areas, where the emotional, linguistic, and intercultural demands of teaching may produce qualitatively different patterns of resilience and adaptation in its unique ways.

Against this backdrop, the present study treats teacher resilience as a multidimensional construct and examines how different resilience configurations are associated with emotional adaptation (i.e., emotion regulation and teacher emotional labour strategy) and professional adaptation (i.e., teacher subjective wellbeing and professional identity) in foreign language education.

## 2 Literature review

### 2.1 Teacher resilience

Teacher resilience entered educational research as an extension of the broader psychological construct of resilience. It became a distinct focus of inquiry in the mid-2000s, most influentially through Gu and Day's (2007) work, and was still characterised by Beltman et al. (2011) as “a relatively recent area of investigation.” Its appeal lies in its explanatory power: resilience helps explain how teachers sustain commitment, effectiveness, and psychological functioning amid the persistent demands of the profession. Importantly, contemporary academia has moved decisively away from treating resilience as a fixed individual trait. Instead, it is conceptualised as multidimensional, socially situated, dynamic, and developmental (Beltman et al., 2011; Gu and Day, 2007; Mansfield et al., 2016). Resilience does not reside solely within the individual teacher but emerges from the ongoing interaction among personal strengths, professional demands, and contextual resources. This relational view is particularly suitable for teaching, where emotional pressures, interpersonal relationships, and institutional expectations jointly and continuously shape how teachers adapt to their work.

Understood ecologically, teacher resilience draws simultaneously on internal resources, such as competence, confidence, optimism, and a sense of meaning, and on external resources embedded in relationships with colleagues, family members, and wider social or spiritual frameworks (Beltman et al., 2011; Mansfield et al., 2016). The four dimensions operationalized in the present study—personal competences, spiritual influences, peer support, and family cohesion (Daniilidou and Platsidou, 2018)—map directly onto this internal-contextual span. Namely, the first two capture intrapersonal strengths and meaning-based resources, whereas the latter two capture relational and contextual support. This multidimensional framing raises a question that has received surprisingly little attention: teachers may differ not only in how much resilience they possess but also in how resilience resources are configured within the individual. Such configural differences are invisible when resilience is collapsed into a single global score.

An adjacent line of inquiry in language teacher psychology, namely, teacher immunity, provides converging evidence for this ecological account. Teacher immunity is conceptualised as a dynamic psychological protection system that may develop in productive or maladaptive forms. Resilience is commonly treated as one component of this broader system rather than as its synonym. Zohrabi and Khalili (2024), for example, found that novice EFL teachers foregrounded external support in their immunity philosophies, whereas experienced teachers placed greater emphasis on internal strengths. In a larger quantitative comparison, Khalili et al. (2024) showed that emotional intelligence, perfectionism, professional development, and job satisfaction predicted immunity among both native and non-native English instructors, while spiritual intelligence was salient only for native teachers, and experience, income, and reflective teaching only for non-native teachers. Cross-cultural evidence further demonstrated that the predictor structure of immunity varied across Iranian, French, and American English teachers. Teaching enjoyment, psychological wellbeing at work, and work engagement were most salient in Iran, whereas emotion regulation, reflection, and self-compassion were more prominent in France and the United States (Khalili et al., 2025). Institutional conditions also mattered. Zohrabi and Paydar (2025) reported higher immunity among public-school than private-institute EFL teachers and negative associations of role overload and job stress with immunity. Although these studies concern teacher immunity rather than resilience directly, they converge on a central premise of the present study. That is, language teachers' adaptive capacity is jointly shaped by personal, emotional, relational, and institutional resources.

This possibility points to a methodological as well as a substantive gap. Research on teacher resilience has been dominated by variable-centred approaches, which model average relations among variables across an entire sample and therefore presuppose population homogeneity. They cannot reveal whether the sample contains subgroups in which resilience resources are organised in qualitatively different ways (Howard and Hoffman, 2018; Morin et al., 2018). Person-centred approaches, by contrast, identify latent subpopulations characterised by distinct configurations of indicators, and are best understood as complementary to, rather than competing with, variable-centred analyses (Marsh et al., 2009). This gap is particularly salient in foreign language education, where the distinctive linguistic, emotional, and intercultural demands of teaching may plausibly generate heterogeneous resilience configurations. Accordingly, teacher resilience needs to be examined not only in terms of overall level but also in terms of its internal configuration across teachers.

Recent person-centred studies reinforce this rationale in teacher research. Sun and Yin (2025) identified four teacher self-efficacy profiles among Chinese teachers, and these profiles differed in affective wellbeing. Li et al. (2025) used multilevel latent profile analysis to show that configurations of job demands, job resources, and personal resources were associated with multidimensional wellbeing among early childhood educators. In the Chinese EFL context, Pan and Wang (2025) identified four teacher AI-literacy profiles and showed that age and teaching experience differentiated profile membership. Although these studies examined constructs other than resilience, they demonstrate that teacher resources and capabilities may be organised in heterogeneous patterns that are obscured by sample-wide averages.

### 2.2 Emotion regulation and teacher emotional labour strategy

Teacher resilience is closely associated with the emotional processes through which teachers respond to professional demands. Teaching is inherently emotional work: teachers must continually manage how emotions are experienced, interpreted, and expressed in classroom and school contexts. In the line of research on emotion regulation, the processes by which individuals influence which emotions they have, when they have them, and how they experience and express them are most commonly represented by two strategies: cognitive reappraisal and expressive suppression (Gross, 1998; Gross and John, 2003). Reappraisal is an antecedent-focused strategy that reframes a situation before the emotional response has fully unfolded, whereas suppression is a response-focused strategy that inhibits emotional expression after the emotion has been activated (Bai and Li, 2025). The two strategies carry markedly different consequences: reappraisal is generally associated with better coping, interpersonal functioning, and wellbeing, whereas habitual suppression tends to be linked to greater strain and poorer adjustment (Gross and John, 2003). These distinctions matter especially in teaching, where emotional demands are persistent, relationally embedded, and publicly visible.

A closely related construct is emotional labour, the regulation of emotional displays in accordance with workplace norms and expectations (Hochschild, 1983). Grandey (2000) explicitly recast emotional labour as emotion regulation enacted in occupational settings, thereby connecting the two concepts: emotion regulation concerns the intrapersonal management of emotional experience, whereas emotional labour concerns the management of emotional display under occupational feeling rules. In teacher research, emotional labour is typically represented by three strategies—surface acting, deep acting, and the expression of naturally felt emotions (Yin, 2012). Surface acting involves displaying emotions that are not genuinely felt or masking genuine ones; deep acting involves modifying inner feelings so that the required display follows authentically; and natural expression reflects the spontaneous display of genuinely felt, role-consistent emotions. Their consequences diverge consistently: surface acting is associated with burnout and lower teaching satisfaction, whereas deep acting and natural expression are linked to healthier adjustment and more positive professional experience (Yin, 2015; Yin et al., 2019). Theoretically, resilience offers a resource-based explanation for these strategic differences. Namely, teachers rich in personal and contextual resources should be better positioned to deploy antecedent-focused and authentic strategies, whereas teachers with depleted resources may be forced back on effortful suppression or surface performance when coping with emotionally demanding situations.

Both lines of research are well developed, but they have largely run in parallel, with attention concentrated on the consequences of particular strategies for burnout, stress, or job satisfaction rather than on their position within teachers' broader adaptive functioning. In particular, little research has examined whether resilience helps explain why some teachers rely predominantly on reappraisal, deep acting, and natural expression while others depend on suppression or surface acting. An integrative examination of emotion regulation and emotional labour in relation to teacher resilience is therefore needed.

Person-centred evidence also suggests that emotional strategies should be considered jointly. Bai and Li (2025) integrated reappraisal, suppression, surface acting, deep acting, and natural expression in a multilevel latent profile analysis of Chinese preschool teachers. Their profiles differed in professional wellbeing and identity, and the coexistence of surface acting and natural expression within one profile showed that apparently contradictory strategies can operate together. In adjacent L2 research, emotional-intelligence profiles have been linked to writing feedback literacy (Long and Zhu, 2025), while academic-emotion profiles have been associated with engagement among Chinese ethnic-minority EFL learners (Liu and Yao, 2026). These findings support a configurational view of affective functioning rather than an exclusive focus on isolated strategy effects.

### 2.3 Teacher subjective wellbeing and professional identity

Beyond emotional processes, teacher resilience should also be reflected in broader indicators of successful adaptation, of which subjective wellbeing and professional identity are arguably the most central. Teacher wellbeing is commonly understood as a multidimensional form of positive psychological functioning in professional life, encompassing affective, relational, and efficacy-related experiences (Hascher and Waber, 2021). Recent theorising has explicitly linked it to resilience within a single integrative model (Hascher et al., 2021). Indicators, such as school connectedness, joy of teaching, and teaching efficacy, capture whether teachers are functioning positively and sustainably at work (Renshaw et al., 2015). Teachers with stronger resilience resources should be better able to cope with stressors, sustain enthusiasm, and preserve a sense of competence. By contrast, weaker resilience leaves teachers more vulnerable to stress, disengagement, and diminished professional satisfaction.

Teacher resilience is also theoretically intertwined with professional identity, namely, teachers' understanding of their role, values, commitment, and sense of belonging within the profession (Beijaard et al., 2004). Dimensions, such as role value, professional value, professional behaviours, and professional belongingness, reflect how teachers position themselves in relation to the profession and the meanings they attach to their work. The relation is plausibly reciprocal: a strong professional identity can serve as a protective resource that supplies continuity, purpose, and motivation under challenging conditions, while resilience supports the enactment and maintenance of a positive identity when teachers must negotiate uncertainty or adversity (Gu and Day, 2007; Pearce and Morrison, 2011). Within the present framework, subjective wellbeing and professional identity are therefore treated as broader indicators of successful professional adaptation, whereas emotion regulation and emotional labour represent the processes through which such adaptation is accomplished in everyday practise.

Although both constructs are widely recognised as central to teachers' professional lives, they have typically been pursued as separate strands of inquiry, and their joint relationship with teacher resilience has received insufficient attention. Little evidence therefore exists on how wellbeing and professional identity together index teachers' successful adaptation, underscoring the need for research that examines them alongside teacher resilience within a unified framework.

Recent profile studies likewise connect teachers' configurations with broader professional adaptation. Zhou et al. (2025) found that pre-service teachers' motivational and coping profiles differed in psychological wellbeing and occupational commitment, while Sun and Yin (2025) documented affective-wellbeing differences across teacher self-efficacy profiles. Bai and Li (2025) reported corresponding differences in professional identity across teacher emotion-regulation profiles. Arslan et al. (2025) further showed that teacher identity itself can form heterogeneous patterns across beliefs, self-efficacy, emotions, motivation, and self-image. These findings suggest that wellbeing and identity are embedded in broader patterns of teachers' resources and functioning rather than being adequately represented by isolated mean-level associations.

Rural teachers may experience distinctive forms of professional vulnerability, including limited instructional resources, fewer opportunities for sustained professional learning, heavier role expectations, and weaker access to specialist support. For foreign language teachers, these contextual constraints may be compounded by the demands of sustaining target-language interaction, providing corrective feedback, and supporting learners' motivation in contexts where exposure to the target language may be limited. However, little is known about how resilience resources are configured among rural foreign language teachers, or whether different resilience configurations are associated with distinct emotional and professional adaptation patterns.

### 2.4 Relationship among teacher resilience, emotional adaptation, and professional adaptation in language education

The relationships among teacher resilience, emotional adaptation, and professional adaptation deserve particular attention in language education. This is because language teachers work in instructional settings where emotion, interaction, identity, and professional functioning are unusually tightly intertwined. Foreign language teaching involves a distinctive combination of demands: sustaining meaningful target-language interaction while managing the balance between the first and target languages (Turnbull and Arnett, 2002), regulating learners' anxiety and motivation, providing continuous corrective feedback, and mediating culturally inflected classroom relationships (Tao et al., 2024). These demands intensify both the emotional and the professional pressures of teaching, making it especially important to consider how resilience operates alongside teachers' emotional and professional functioning rather than as an isolated construct.

Within this broader system, teacher resilience may be viewed as a foundational adaptive resource, whereas emotion regulation and emotional labour strategy constitute the adaptive processes through which everyday emotional demands are managed. Emotion regulation captures how teachers manage internal emotional experience (Gross and John, 2003), while emotional labour strategy reflects how they regulate emotional displays in accordance with professional expectations (Hochschild, 1983; Yin, 2012). Because language teaching is highly interactional and emotionally demanding, these processes are likely to be especially salient in language classrooms, where teachers must continuously respond to students' affective needs, sustain target-language interaction, and manage the emotional demands of pedagogical performance.

At the same time, teacher subjective wellbeing and professional identity may be regarded as broader indicators of successful professional adaptation. Subjective wellbeing reflects whether teachers are functioning positively and sustainably in their work (Renshaw et al., 2015), whereas professional identity concerns how teachers understand their role, values, commitment, and belonging within the profession (Beijaard et al., 2004). In language education, where teachers work at the intersection of pedagogy, language use, and intercultural communication, these two domains are especially consequential for whether teachers can maintain enthusiasm, competence, and a stable sense of self under demanding conditions.

Taken together, the constructs reviewed above are conceptually distinct but theoretically interconnected. In language education, however, they have tended to be examined piecemeal, one or two links at a time. Consequently, limited evidence is available on how teacher resilience, emotional adaptation, and professional adaptation operate together within a unified framework in foreign language education, especially in rural areas. This unresolved question provides the rationale for the present study.

### 2.5 The present study

Against this backdrop, the present study focuses specifically on junior secondary foreign language teachers working predominantly in rural and rural-adjacent schools in Fujian Province, China. In these settings, teachers may experience uneven access to subject-specific professional development, instructional resources, specialist support, and wider professional networks, while remaining responsible for sustaining target-language interaction, regulating learners' affect, providing corrective feedback, and meeting institutional and community expectations. These contextual conditions make teacher resilience particularly consequential and render an ecological perspective, one that considers both personal and relational resources, especially appropriate.

Within this rural foreign language education context, teacher resilience is conceptualised as a multidimensional construct comprising personal competences, spiritual influences, peer support, and family cohesion (Daniilidou and Platsidou, 2018). The study examines how these resilience resources are associated with emotional adaptation (emotion regulation and emotional labour strategy) and professional adaptation (subjective wellbeing and professional identity). Emotion regulation and emotional labour strategy are treated as proximal emotional-management processes because they reflect how teachers manage internal emotional experiences and occupational display requirements. Subjective wellbeing and professional identity are regarded as broader indicators of whether teachers are functioning positively and sustainably in their professional roles. Teacher resilience is therefore positioned theoretically as a foundational adaptive resource associated with both emotional management and professional functioning.

To address the limitations identified above, the study combines variable-centred and person-centred perspectives, which provide complementary forms of evidence (Howard and Hoffman, 2018; Marsh et al., 2009; Morin et al., 2018). The variable-centred component examines the latent associations among the four resilience dimensions and the emotional and professional adaptation domains, clarifying whether stronger resilience resources are generally associated with more favourable functioning among rural foreign language teachers. The person-centred component employs latent profile analysis (Spurk et al., 2020) to investigate whether teachers working within broadly similar rural and rural-adjacent educational conditions nevertheless exhibit distinct configurations of personal and relational resilience resources. This distinction is important because rural teachers should not be treated as a homogeneous group: limited contextual resources may coexist with strong family cohesion or peer support for some teachers, whereas others may possess strong personal competences but weaker relational support. Although recent latent-profile research in language education has revealed meaningful heterogeneity in learners' motivation, engagement, and emotional functioning (Lei and Ye, 2025), comparable evidence concerning the resilience configurations of rural foreign language teachers remains limited.

By integrating these constructs within a single framework, the study seeks to make three contributions. First, it extends teacher resilience research into the comparatively under examined context of rural foreign language education, where linguistic and emotional teaching demands intersect with potentially uneven professional and institutional resources. Second, it advances the ecological understanding of resilience by examining how personal, meaning-based, peer, and family resources are configured within individual rural teachers rather than reducing resilience to a single overall score. Third, it provides a differentiated evidence base for rural teacher support by identifying which resilience configurations are associated with more or less favourable emotional and professional adaptation. Such evidence may inform context-sensitive professional development, mentoring, school-based collegial communities, and regional or online support networks for rural foreign language teachers.

Based on the literature reviewed above, the study asks:

RQ1. What latent profiles of teacher resilience emerge among rural foreign language teachers on the basis of personal competences, spiritual influences, peer support, and family cohesion?
RQ2. To what extent do the identified teacher resilience profiles differ in emotional adaptation (emotion regulation and emotional labour strategy) and professional adaptation (subjective wellbeing and professional identity)?

## 3 Methods

### 3.1 Research design

The study adopted a cross-sectional survey design to examine the relationships among teacher resilience, emotional adaptation and professional adaptation, and to identify latent subgroups of teachers based on their resilience configurations. Consistent with the conceptual framework developed in the literature review, teacher resilience was treated as a multidimensional construct comprising personal competences, spiritual influences, peer support, and family cohesion. The design further incorporated two emotion regulation strategies (reappraisal and suppression), three emotional labour strategies (surface acting, deep acting, and natural expression), three indicators of subjective wellbeing (school connectedness, joy of teaching, and teaching efficacy), and four dimensions of professional identity (role value, professional value, professional behaviours, and professional belongingness), yielding 16 substantive dimensions in total. Methodologically, the study combined variable-centred and person-centred analyses (Morin et al., 2018) so that both the overall nomological structure among the constructs and the within-sample heterogeneity in resilience configurations could be examined within a single analytic framework.

### 3.2 Population, sampling, and procedure

The target population comprised in-service junior secondary English teachers working in rural or rural-adjacent schools in Fujian Province, China. A non-probability convenience sampling strategy with voluntary response was used. The online survey link was circulated through these professional communication channels, and teachers chose independently whether to participate. Eligibility required current service as a junior secondary English teacher in Fujian Province. Rurality was operationalized by school administrative location: district/township- and county-level schools were treated as rural or rural-adjacent, whereas municipal-level schools were viewed as urban. The sample size was determined by the number of eligible, usable voluntary responses obtained during the survey period rather than by a finite-population formula.

Data were collected online through a widely used Chinese survey platform Wenjuanxing (https://www.wjx.cn). The questionnaire was administered in Chinese to ensure clarity and accessibility. All participants completed the questionnaire. Participation was voluntary, informed consent was obtained before the questionnaire began, and anonymity was guaranteed. A total of 262 eligible and usable responses were retained for analysis.

### 3.3 Measures

All constructs were assessed with self-report instruments. The questionnaire comprised 79 items loading on 16 first-order dimensions organised into five domains: teacher resilience, emotion regulation, teacher emotional labour strategy, teacher subjective wellbeing, and teacher professional identity. All items were rated on five-point Likert scales, with higher scores indicating higher standing on the corresponding construct. Internal consistency estimates (Cronbach's α) reported below were computed from the present analytic sample (*N* = 262).

Teacher resilience was measured with the 26-item Teachers' Resilience Scale (TRS; Daniilidou and Platsidou, 2018), which assesses four dimensions: personal competences (9 items; α = 0.90), spiritual influences (3 items; α = 0.57), peer support (8 items; α = 0.91), and family cohesion (6 items; α = 0.93). The items capture teachers' perceived ability to adapt to change, remain focused under pressure, and cope with challenges; the strength they draw from spiritual or value-based beliefs; the support they receive from peers; and the cohesion of their family relationships. These four dimensions served as the profile indicators in the latent profile analysis.

Emotion regulation was measured with the 10-item Emotion Regulation Questionnaire (ERQ; Gross and John, 2003), which assesses the habitual use of cognitive reappraisal (6 items; α = 0.96) and expressive suppression (4 items; α = 0.81). Reappraisal refers to construing a potentially emotion-eliciting situation in a way that changes its emotional impact, whereas suppression refers to inhibiting ongoing emotion-expressive behaviour after an emotion has been activated.

Teacher emotional labour strategy was measured with the 13-item Teacher Emotional Labour Strategy Scale (TELSS; Yin, 2012), adapted and validated for teachers in China. The TELSS assesses surface acting (6 items; α = 0.96), the faking or masking of emotions to display what the job requires; deep acting (4 items; α = 0.90), the attempt to modify inner feelings so that the desired display follows; and the expression of naturally felt emotions (3 items; α = 0.91), the spontaneous display of genuinely felt, role-consistent emotions.

Teacher subjective wellbeing was assessed with a 12-item measure informed by the Teacher Subjective Wellbeing Questionnaire (TSWQ; Renshaw et al., 2015), represented here by three indicators: school connectedness (4 items; α = 0.90), joy of teaching (4 items; α = 0.95), and teaching efficacy (4 items; α = 0.90). The TSWQ was initially developed around these three teacher-specific indicators, although its final validated structure retained school connectedness and teaching efficacy as the core factors; consistent with the analytic model adopted here, all three indicators were used, and items were administered on five-point scales.

Teacher professional identity was measured with an 18-item Teacher Professional Identity Scale assessing four dimensions: role value (7 items; α = 0.89), professional value (4 items; α = 0.92), professional behaviours (4 items; α = 0.89), and professional belongingness (3 items; α = 0.74). The items capture teachers' perceptions of the social significance of teaching, their pride in being a teacher, their profession-consistent behaviours, and their sense of belonging to the teaching profession.

### 3.4 Data analysis

This study conducted a series of data analyses, including descriptive statistics, confirmatory factor analysis (CFA), latent profile analysis (LPA), and profile comparisons with omnibus Wald tests.

Firstly, descriptive statistics, namely, means, standard deviations, skewness, and kurtosis, were computed using Mplus 8.80 to examine the distributional properties of the 16 study dimensions and to provide a preliminary assessment of normality.

Secondly, a CFA was conducted using Mplus 8.80 to evaluate the adequacy of the measurement model, in which the 16 dimensions were specified as distinct but correlated latent factors. Because all items were rated on five-point Likert scales, the indicators were treated as ordered categorical variables and the model was estimated with the robust weighted least squares estimator (WLSMV). Model fit was assessed using the chi-square statistic, the comparative fit index (CFI), the Tucker–Lewis index (TLI), the standardised root mean square residual (SRMR), and the root mean square error of approximation (RMSEA). The model fit the data well, χ^2^(2,882) = 4213.078, *p* < 0.001, CFI = 0.987, TLI = 0.986, SRMR = 0.062, RMSEA = 0.043, and latent correlations among the 16 dimensions were extracted from this model to examine the interrelations between teacher resilience and the four adaptation domains.

Then, LPA was conducted using Mplus 8.80 to explore teachers' resilience profiles, with the four resilience dimensions—personal competences, spiritual influences, peer support, and family cohesion—serving as profile indicators. The indicators were *z*-standardised prior to estimation, and models with one to five profiles were estimated under the conventional specification in which indicator variances are held equal across profiles and within-profile covariances are fixed at zero; multiple sets of random starting values were used to avoid convergence on local maxima. Following established practise in person-centred mixture modelling (Nylund et al., 2007; Nylund-Gibson and Choi, 2018; Sinha et al., 2021; Spurk et al., 2020), the competing models were evaluated using multiple criteria to determine model fit and classification quality. The Akaike information criterion (AIC), the Bayesian information criterion (BIC), and the sample-size-adjusted BIC (SBIC) were used to assess model fit, with lower values indicating a better-fitting model. The Vuong–Lo–Mendell–Rubin likelihood ratio test (VLMR-LRT) and the bootstrapped likelihood ratio test (BLRT) were employed to compare models with k and k-−1 profiles; a significant *p*-value suggests that the model with k profiles provides a better fit. Entropy, which ranges from 0 to 1, was used to evaluate classification accuracy, with values closer to 1 indicating more precise classification of individuals into profiles. In addition, parsimony, interpretability, and the size of the smallest profile were taken into account in model selection.

Finally, the identified profiles were interpreted on the basis of their standardised indicator means and were compared on the twelve adaptation variables—reappraisal, suppression, surface acting, deep acting, natural expression, school connectedness, joy of teaching, teaching efficacy, professional value, role value, professional behaviours, and professional belongingness. These comparisons were based on model-estimated means and standard errors together with omnibus Wald χ^2^ tests, which account for classification uncertainty more adequately than assigning individuals to profiles and comparing observed group means (Asparouhov and Muthén, 2014; Bai and Li, 2025).

## 4 Results

### 4.1 Participant characteristics

The analytic sample comprised 262 in-service junior secondary English teachers. The sample was predominantly female (*n* = 214, 81.7%; male *n* = 48, 18.3%). In terms of age, 8.0% of respondents were 18–25 years old, 10.7% were 26–30, 22.9% were 31–40, 39.3% were 41–50, and 19.1% were 51–60. Teaching experience ranged from 0 to 35 years (*M* = 18.75, SD = 11.45). Most held a bachelor's degree (*n* = 239, 91.2%), with smaller numbers holding a college diploma (5.7%), a postgraduate degree (2.3%), or a junior-secondary qualification (0.8%). Participants worked predominantly in district/township-level (*n* = 197, 75.2%) or county-level schools (*n* = 65, 24.8%). All participants are native speakers of Chinese. Table 1 provides the complete demographic and professional profile of the respondents.

**Table 1:** Participant characteristics.

Characteristic	Category/statistic	*n* (%)
Gender	Female	214 (81.7)
	Male	48 (18.3)
Age (years)	18–25	21 (8.0)
	26–30	28 (10.7)
	31–40	60 (22.9)
	41–50	103 (39.3)
	51–60	50 (19.1)
English teaching experience	*M* = 18.75; SD = 11.45; range = 0–35 years	262
Academic degree	Bachelor's degree	239 (91.2)
	College diploma	15 (5.7)
	Postgraduate degree	6 (2.3)
	Junior-secondary qualification	2 (0.8)
School administrative level	District/township	197 (75.2)
	County	65 (24.8)
Geographic coverage	Eight of Fujian's nine prefecture-level cities	262 (100)
First language (L1)	Chinese	262 (100)

Percentages may not total 100 because of rounding.

### 4.2 Preliminary analyses: descriptive statistics, normality, and confirmatory factor analysis

Table 2 shows the descriptive statistics for teacher resilience, emotion regulation, teacher emotional labour strategy, subjective wellbeing, and professional identity. The CFA demonstrated a good fit to the data, χ^2^ (2,882) = 4,213.078, *p* < 0.001, CFI = 0.987, TLI = 0.986, SRMR = 0.062, and RMSEA = 0.043. The skewness and kurtosis values were small in magnitude and approximately within ± 1 (skewness = −0.53 to 0.45; kurtosis = −0.90 to 0.43), indicating no serious univariate-normality concerns.

**Table 2:** Descriptive statistics for substantive variables.

Variable	*M*	SD	Skewness	Kurtosis
**Teacher resilience**				
1 Personal competences	3.72	0.64	−0.03	−0.32
2 Spiritual influences	3.61	0.65	0.16	0.31
3 Peer support	3.69	0.67	0.33	−0.37
4 Family cohesion	4.10	0.67	−0.43	−0.32
**Emotion regulation**				
5 Reappraisal	3.94	0.69	−0.21	−0.42
6 Suppression	3.28	0.72	0.48	0.35
**Teacher emotional labour strategy**				
7 Surface acting	2.54	1.00	0.32	−0.33
8 Deep acting	3.50	0.83	−0.28	0.43
9 Natural expression	3.90	0.75	−0.12	−0.61
**Teacher subjective wellbeing**				
10 School connectedness	3.81	0.79	−0.04	−0.90
11 Joy of teaching	3.83	0.78	0.04	−0.90
12 Teaching efficacy	3.69	0.67	0.39	−0.54
**Teacher professional identity**				
13 Professional value	4.22	0.68	−0.53	−0.43
14 Role value	3.83	0.68	−0.02	−0.39
15 Professional behaviours	4.22	0.62	−0.45	−0.45
16 Professional belongingness	4.03	0.65	−0.19	−0.54

*M* = mean. SD = standard deviation.

### 4.3 Preliminary latent correlations among the study variables

Table 3 presents the latent correlations among the 16 study dimensions. Most associations were statistically significant and theoretically coherent. Surface acting showed the weakest and least consistent relationships with the other dimensions. It was nevertheless retained because of its theoretical importance to the emotional-labour framework and its potential to contribute uniquely to the subsequent profile comparisons.

**Table 3:** Latent correlations from CFA.

	PC	SI	PS	FC	RE	SU	SA	DA	NE	SC	JT	TE	RV	PV	PBH	PBL
PC	1															
SI	0.57^***^	1														
PS	0.68^***^	0.51^***^	1													
FC	0.56^***^	0.41^***^	0.67^***^	1												
RE	0.70^***^	0.54^***^	0.73^***^	0.71^***^	1											
SU	0.42^***^	0.36^***^	0.34^***^	0.35^***^	0.42^***^	1										
SA	−0.1	0.19^**^	0.00	−0.06	−0.11	0.40^***^	1									
DA	0.32^***^	0.24^***^	0.40^***^	0.37^***^	0.42^***^	0.30^***^	0.08	1								
NE	0.54^***^	0.33^***^	0.54^***^	0.50^***^	0.64^***^	0.26^***^	−0.31^***^	0.58^***^	1							
SC	0.56^***^	0.37^***^	0.60^***^	0.63^***^	0.62^***^	0.36^***^	−0.16^*^	0.33^***^	0.58^***^	1						
JT	0.64^***^	0.44^***^	0.59^***^	0.59^***^	0.67^***^	0.33^***^	−0.17^**^	0.40^***^	0.64^***^	0.76^***^	1					
TE	0.64^***^	0.46^***^	0.58^***^	0.58^***^	0.61^***^	0.43^***^	−0.04	0.40^***^	0.63^***^	0.67^***^	0.75^***^	1				
RV	0.56^***^	0.40^***^	0.52^***^	0.57^***^	0.60^***^	0.36^***^	−0.09	0.38^***^	0.65^***^	0.59^***^	0.63^***^	0.65^***^	1			
PV	0.53^***^	0.38^***^	0.49^***^	0.58^***^	0.62^***^	0.22^***^	−0.20^**^	0.43^***^	0.61^***^	0.71^***^	0.74^***^	0.73^***^	0.75^***^	1		
PBH	0.49^***^	0.38^***^	0.50^***^	0.61^***^	0.63^***^	0.26^***^	−0.14^*^	0.35^***^	0.62^***^	0.57^***^	0.58^***^	0.60^***^	0.86^***^	0.66^***^	1	
PBL	0.55^***^	0.40^***^	0.53^***^	0.60^***^	0.65^***^	0.33^***^	−0.09	0.42^***^	0.63^***^	0.65^***^	0.68^***^	0.67^***^	0.82^***^	0.82^***^	0.79^***^	1

^***^p < 0.001, ^**^p < 0.01, ^*^p < 0.05.

### 4.4 Results for Research Question 1: Latent teacher resilience profiles

*RQ1. What latent profiles of teacher resilience emerge among rural foreign language teachers on the basis of personal competences, spiritual influences, peer support, and family cohesion?*

**Figure 1:**
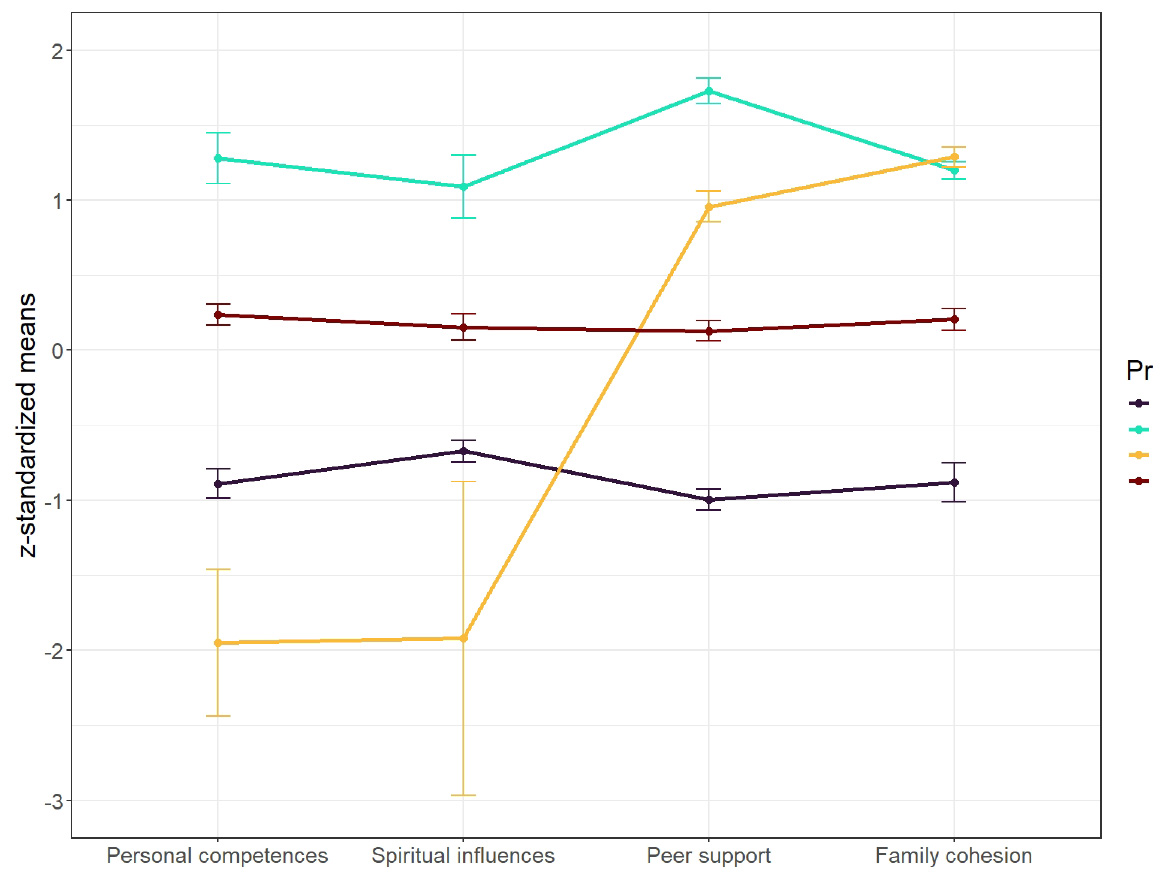
Latent profiles of teachers resilience based on standardised means.

To answer RQ1, Table 4 summarises the model-comparison results for the LPA. AIC, BIC, and SBIC values all declined steadily as more profiles were added, and every BLRT *p*-value fell below 0.001—signs that adding profiles enhanced model fit. The VLMR test, however, lost significance once a fifth profile was introduced (*p* = 0.2216), indicating no meaningful gain from that additional profile. By contrast, in the four-profile model both the VLMR (*p* < 0.001) and BLRT (*p* < 0.001) tests reached significance, and entropy exceeded.8, pointing to the four-profile solution as the most parsimonious and best-fitting representation of the data. As shown in Figure 1, the four groups were named “Frail” (lowest levels of peer support and family cohesion, but higher levels of personal competences and spiritual influence than profile 3; profile 1, *n* = 83), “Hardcore” (highest levels of personal competences and spiritual influence, even a higher level of peer support, but a lower level of family cohesion than profile 3; profile 2, *n* = 38), “Outreaching” (high levels of peer support and family cohesion, but lowest levels of personal competences and spiritual influence; profile 3, *n* = 14), and “Balanced” (similar levels of personal competences, spiritual influence, peer support, and family cohesion, with the first two higher than profiles 3 and 4, and the rest two lower than profiles 1 and 4; profile 4, *n* = 127), respectively.

**Table 4:** Fit indices for models with different number of latent profiles.

Number of profiles	AIC	BIC	SBIC	Entropy	VLMR LRT *p*-values	BLRT *p*-values	Profile sizes
1	2,883.924	2,912.191	2,886.829	–	–	–	262
2	2,621.899	2,667.833	2,626.621	0.747	0.0019	< 0.001	141/121
3	2,473.176	2,536.77	2,479.714	0.871	< 0.001	< 0.001	140/84/38
**4**	**2,455.389**	**2,536.657**	**2,463.742**	**0.899**	**< 0.001**	**< 0.001**	**127/83/38/14**
5	2,438.376	2,537.311	2,448.546	0.889	0.2216	< 0.001	129/48/45/29/11

The black bold font represents the chosen optimal model.

### 4.5 Results for Research Question 2: Profile differences in emotional and professional adaptation

*RQ2. To what extent do the identified teacher resilience profiles differ in emotional adaptation (emotion regulation and emotional labour strategy) and professional adaptation (subjective wellbeing and professional identity)?*

**Figure 2:**
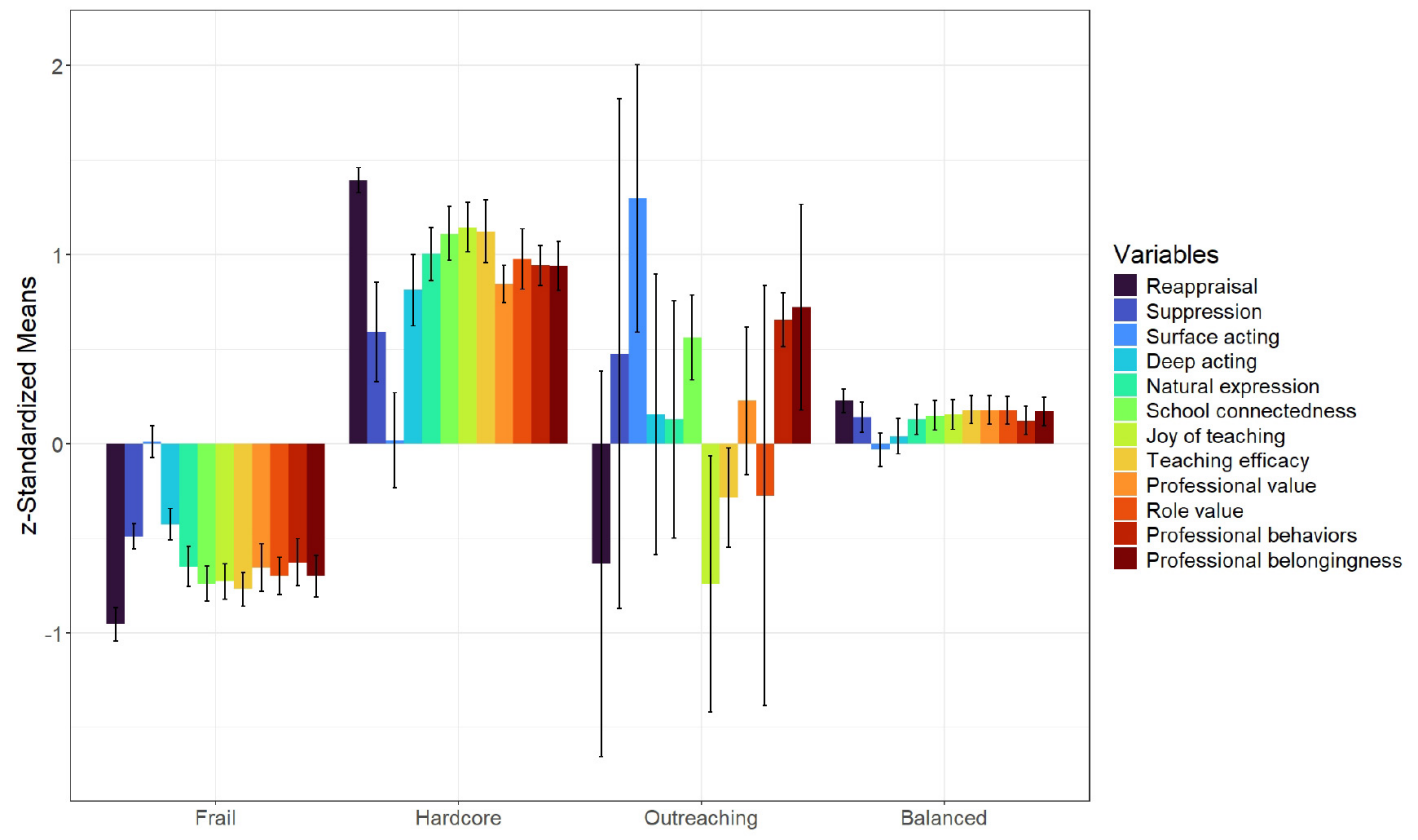
*Z*-standardised patterns of adaptive emotional processes and functioning outcomes across teacher resilience profiles.

To answer RQ2, Table 5 reports differences across the four teacher resilience profiles, namely, Frail, Hardcore, Outreaching, and Balanced, on twelve adaptive emotional and functioning outcomes, giving each profile's mean and standard error and an overall Wald χ^2^ test. Figure 2. shows the same outcomes as z-standardised means. Eleven of the twelve outcomes differed significantly across profiles (all *p* < 0.001); only surface acting was non-significant (χ^2^ = 3.479, *p* =0.324). Differences were largest for reappraisal (χ^2^ = 470.947), joy of teaching (χ^2^ = 141.476), and school connectedness (χ^2^ = 133.055).

**Table 5:** Differences in adaptive emotional processes and functioning outcomes across teacher resilience profiles.

Variable	Frail		Hardcore		Outreaching		Balanced		Wald test	
	*M*	*(SE)*	*M*	*(SE)*	*M*	*(SE)*	*M*	*(SE)*	χ^2^	*P*
Reappraisal	3.278	(0.061)	4.908	(0.046)	3.500	(0.707)	4.098	(0.043)	470.947	0.000
Suppression	2.929	(0.049)	3.709	(0.190)	3.625	(0.973)	3.383	(0.058)	43.549	0.000
Surface acting	2.550	(0.085)	2.555	(0.251)	3.834	(0.707)	2.507	(0.090)	3.479	0.324
Deep acting	3.141	(0.069)	4.173	(0.156)	3.625	(0.619)	3.529	(0.077)	41.590	0.000
Natural expression	3.414	(0.079)	4.659	(0.106)	4.000	(0.472)	4.000	(0.059)	90.627	0.000
School connectedness	3.218	(0.074)	4.686	(0.113)	4.250	(0.177)	3.923	(0.061)	133.055	0.000
Joy of teaching	3.261	(0.074)	4.727	(0.102)	3.250	(0.531)	3.950	(0.060)	141.476	0.000
Teaching efficacy	3.174	(0.060)	4.449	(0.111)	3.500	(0.177)	3.814	(0.049)	125.469	0.000
Professional value	3.777	(0.085)	4.794	(0.068)	4.375	(0.265)	4.343	(0.051)	88.930	0.000
Role value	3.351	(0.067)	4.496	(0.108)	3.643	(0.758)	3.950	(0.051)	93.746	0.000
Professional behaviours	3.830	(0.077)	4.804	(0.065)	4.625	(0.088)	4.295	(0.046)	105.367	0.000
Professional belongingness	3.574	(0.071)	4.641	(0.085)	4.500	(0.354)	4.141	(0.049)	96.421	0.000

Profiles were derived from the four teacher resilience indicators: personal competences, spiritual influences, peer support, and family cohesion. The variables listed in the rows were treated as distal outcomes or associated adaptive functioning indicators. Wald χ^2^ tests were used to examine overall profile differences. *P* values reported as 0.000 should be interpreted as *p* < 0.001.

The dominant pattern was a resilience gradient: Hardcore teachers scored highest and Frail teachers lowest, with Balanced teachers in between, near the sample average. Reappraisal, for instance, rose from 3.278 (Frail) to 4.098 (Balanced) to 4.908 (Hardcore), and the same ordering held for deep acting, natural expression, and all professional-identity measures. Figure 2 shows this clearly—Frail bars all fall below the mean, Hardcore bars well above, and Balanced hugs the zero line. The Outreaching profile was the exception, behaving unevenly and with marked uncertainty (its standard errors are several times larger than other profiles', reflecting its relatively small size, n = 14). It was lowest on joy of teaching (3.250) and low on reappraisal and teaching efficacy, yet comparatively high on professional behaviours, professional belongingness, and surface acting (3.834, the highest of any profile).

Overall, higher resilience was associated with more adaptive emotion regulation and stronger professional functioning, with surface acting the only outcome unrelated to resilience. Outreaching appeared qualitatively distinct, seemingly relying on surface acting and relational belonging despite low personal resources.

## 5 Discussion

The present study set out to examine teacher resilience as a multidimensional construct in foreign language education, combining variable-centred and person-centred analyses to address two research questions: whether distinct resilience profiles could be identified among rural foreign language teachers (RQ1), and whether such profiles differed in emotional adaptation (emotion regulation and emotional labour strategy) and professional adaptation (subjective wellbeing and professional identity) (RQ2). Four latent profiles (Frail, Hardcore, Outreaching, and Balanced) emerged and these profiles showed substantial and largely systematic differences across eleven of the twelve adaptive outcomes examined. Taken together, the findings support the view of teacher resilience as a foundational adaptive resource (Gu and Day, 2007; Mansfield et al., 2016) while also revealing meaningful heterogeneity in how resilience resources are configured within individual teachers. This section discusses the two sets of findings in turn.

### 5.1 Four resilience profiles among foreign language teachers (RQ1)

With respect to RQ1, the latent profile analysis identified four interpretable teacher resilience profiles. Three of these profiles—Frail, Balanced, and Hardcore—were differentiated primarily by overall level, forming an ordered continuum from consistently low, through average, to consistently high standing across the four resilience dimensions. The remaining Outreaching profile was differentiated more by shape than by level, reflecting a more uneven configuration of resilience resources. The predominance of the three level-based profiles, which together accounted for approximately 95% of the sample, suggests that for most foreign language teachers, personal competences, spiritual influences, peer support, and family cohesion tend to rise and fall together. This finding is consistent with the positive latent correlations among the four resilience dimensions observed in the variable-centred analysis. It also echoes a common pattern in person-centred teacher research, where profiles differing in elevation rather than shape often dominate.

Comparable profile structures have been reported in recent teacher research. Pan and Wang (2025) found a predominantly ordered Poor-to-Excellent gradient in Chinese EFL teachers' AI literacy, while Sun and Yin (2025) identified a broad teacher self-efficacy gradient alongside a smaller Student-Insecure profile marked by a specific weakness in efficacy for student engagement. Li et al. (2025) likewise demonstrated that job, personal, and contextual resources can combine into differentiated profiles with implications for wellbeing. The present findings extend this emerging literature by showing that the personal, meaning-based, peer, and family resources constituting teacher resilience can display both a dominant elevation gradient and a smaller crossover configuration in rural foreign language education.

These findings both converge with and extend recent research on language teacher immunity, a related but broader construct concerned with teachers' psychological protection against professional stressors. Zohrabi and Khalili (2024) found that novice EFL teachers foregrounded external support, whereas experienced teachers emphasised internal confidence and emotion-management skills. The present Frail–Balanced–Hardcore gradient is compatible with the joint variation of internal and relational resources, while the Outreaching pattern conceptually resembles the externally anchored orientation reported among novice teachers. However, the present study differs in that the profiles were derived empirically from four resilience dimensions within a single rural teacher sample rather than imposed through predefined career-stage groups. Likewise, Khalili et al. (2024) showed that the predictors of teacher immunity differed between native and non-native English instructors, and Khalili et al. (2025) identified different predictor patterns across Iran, France, and the United States. Those between-group findings support the context-sensitive character of teacher adaptation; the current study adds a within-context perspective by showing that marked heterogeneity can also occur among teachers who share the same broad regional and occupational setting.

Interpreted in the context of rural foreign language education, these findings are particularly meaningful. Rural teachers' resilience resources are likely to be shaped by uneven access to professional learning opportunities, school-level support, and collegial networks. The Frail profile suggests that a substantial group of teachers may experience depleted resources across both internal and relational domains. At the same time, the roles of peer support and family cohesion highlight the ecological nature of teacher resilience in resource-constrained settings. In this sense, resilience should not be understood solely as an individual psychological capacity, but as a configuration of personal, relational, and contextual resources.

However, the four-profile solution was not solely level-based. The Outreaching profile (n = 14) showed a distinctive crossover configuration. Specifically, it had the lowest standing on personal competences and spiritual influences, but above-average peer support and high family cohesion. Although relatively small, this profile is conceptually informative because it shows that internal and relational resilience resources do not always co-occur within individual teachers. In other words, some teachers may report limited personal or meaning-based resources while still drawing on strong relational support from colleagues and family.

This configuration would be difficult to detect through variable-centred analyses alone, because the consistently positive correlations among the resilience dimensions suggest that these resources generally rise and fall together at the sample level. The person-centred analysis therefore added important discriminating power by revealing not only a dominant resilience gradient, but also a minority configuration in which relational resources were stronger than internal resources. This finding supports the multidimensional and ecological view of teacher resilience, which argues that resilience does not reside solely within the individual but emerges from the interaction between personal strengths and contextual resources (Beltman et al., 2011; Gu and Day, 2007; Mansfield et al., 2016).

This pattern may be especially relevant to rural foreign language education, where teachers' access to professional learning opportunities and school-level support may be uneven. In such contexts, peer support and family cohesion may partly compensate for weaker internal resilience resources and help sustain teachers' professional functioning.

### 5.2 Profile differences in adaptive emotional processes and functioning outcomes (RQ2)

With respect to RQ2, the four profiles differed significantly on eleven of the twelve outcomes, and the dominant pattern was a clear resilience gradient. Specifically, Hardcore teachers reported the most adaptive functioning, Frail teachers the least, with Balanced teachers consistently in between. The gradient held across emotion regulation, particularly reappraisal, all two aspects of emotional labour, all three indicators of subjective wellbeing, and all four dimensions of professional identity. These findings provide direct support for the framework developed in the literature review, in which teacher resilience was conceptualised as a foundational adaptive resource supporting both the emotional processes and the professional outcomes of teaching. Teachers rich in personal and contextual resources appear better equipped to reframe emotionally demanding situations, align inner feelings with professional displays through deep acting or genuine expression, sustain wellbeing, and maintain a positive professional identity (Gross and John, 2003; Yin et al., 2019). This resilience gradient may be particularly consequential in rural foreign language teaching contexts, where teachers' access to sustained professional development, specialist support, and collegial learning opportunities may be uneven. In such contexts, resilience resources may play an especially important role in sustaining both emotional and professional functioning.

The overall gradient is consistent with profile-outcome patterns reported elsewhere. Bai and Li (2025) found that Chinese preschool teachers in Adaptive and Deep emotion-regulation profiles showed more favourable professional functioning than those in the Strained profile. Sun and Yin (2025) likewise observed the highest contentment and enthusiasm in the Globally High Self-Efficacy profile and the least favourable affective wellbeing in the Extremely Low Self-Efficacy profile, while Zhou et al. (2025) linked the High Functioning pre-service teacher profile to stronger psychological wellbeing and occupational commitment. These convergent findings suggest that profiles characterised by richer personal and regulatory resources tend to be accompanied by more favourable emotional and professional functioning across different teacher populations.

The magnitude of the differences is also instructive. The largest profile effects emerged for cognitive reappraisal, joy of teaching, and school connectedness. That reappraisal showed by far the strongest differentiation suggests that the capacity to cognitively reconstrue demanding situations may be the emotional process most tightly coupled to teachers' resilience resources, consistent with its established status as an adaptive antecedent-focused strategy (Gross, 1998; Gross and John, 2003). The strong differentiation of joy of teaching and school connectedness further indicates that resilience is closely bound up with teachers' experienced positivity and belonging at work, in line with conceptualizations of teacher wellbeing as positive psychological functioning in professional life (Hascher and Waber, 2021; Renshaw et al., 2015). For rural foreign language teachers, these two wellbeing indicators may be especially important. When external instructional resources and professional learning opportunities are less readily available, teachers' sense of belonging to the school community and their ability to preserve joy in teaching may become central psychological resources that help sustain professional adaptation. The pattern also accords with recent EFL evidence linking emotional intelligence and motivation to job satisfaction (Zohrabi and Maleki, 2025) and showing that emotion regulation, teaching enjoyment, psychological wellbeing at work, and work engagement can differentiate teacher immunity across cultural settings (Khalili et al., 2025).

Interestingly, suppression also increased across the resilience gradient, with Hardcore teachers reporting the highest levels. Although suppression is often characterised as maladaptive in Western samples (Gross and John, 2003), its positive association with resilience here may reflect the relational and normative texture of Chinese school contexts, where restraint in emotional expression can serve interpersonal harmony and professional appropriateness. This interpretation may be particularly relevant in rural schools, where interpersonal relationships within school communities can be close and where teachers may be especially attentive to maintaining harmony with colleagues, students, and parents. Resilient teachers may thus command a broader and more flexible regulatory repertoire—including strategic restraint—rather than simply avoiding response-focused regulation. This interpretation converges with research on Chinese teachers' emotional labour suggesting that the adaptiveness of particular strategies is conditioned by cultural display rules (Yin, 2012, 2015). Bai and Li (2025) similarly found that Chinese teachers could combine strategies conventionally treated as inconsistent, including surface acting and natural expression, reinforcing the view that regulatory adaptiveness may be configurational and culturally contingent.

Surface acting was the sole outcome on which the profiles did not differ. This null finding is itself informative. Whereas reappraisal, deep acting, and natural expression scaled with teachers' resilience resources, the faking or masking of emotions appears to be governed less by personal resources than by situational display requirements that bear on all teachers alike (Grandey, 2000; Hochschild, 1983). In other words, surface acting may function as a context-driven, job-imposed strategy rather than a resource-dependent one. It is a reading consistent with its weak correlations with the other study variables in the variable-centred analysis and with prior evidence that surface acting follows occupational feeling rules more than individual dispositions (Yin, 2015). In rural teaching contexts, such display rules may be intensified by teachers' visible social roles within relatively close school communities.

Finally, the Outreaching profile departed from the gradient in a theoretically suggestive way. Despite reporting high family cohesion and above-average peer support, these teachers showed the lowest joy of teaching, low reappraisal and teaching efficacy, and yet comparatively high professional behaviours and professional belongingness, together with the highest surface acting of any profile. This configuration is consistent with a compensatory mode of adaptation. Namely, lacking internal resources of competence and meaning, such teachers may sustain their professional membership through relational anchoring and effortful display management, performing the emotional requirements of the role even as its intrinsic rewards remain out of reach. An analogous dissociation between behavioural and emotional functioning was also reported by Lin and Liu (2025). In their study, beginning teachers with high autonomy support and adaptability demonstrated the strongest innovative work behaviour, yet experienced levels of stress comparable to those of a low-resource profile when time pressure remained elevated. The findings suggest that favourable personal and contextual resources may sustain professional behaviour without uniformly protecting teachers from emotional strain. Similarly, Bai and Li (2025) found that Chinese teachers could combine apparently discrepant emotion-regulation strategies within the same profile. For example, their Strained profile combined elevated surface acting with natural expression. They further suggested that professional belongingness may depend partly on social connexions and collaborative school culture rather than on individual emotion regulation alone.

This pattern may be especially plausible in rural foreign language education, where teachers' professional lives are often embedded in close interpersonal and community relationships. Peer support and family cohesion may provide important relational anchors when individual competence or meaning-based resources are weaker. Meanwhile, the pattern also suggests that relational support alone may not be sufficient to secure positive inner functioning, particularly joy of teaching, reappraisal, and teaching efficacy.

## 6 Conclusion, implications, and limitations

### 6.1 Conclusion

Drawing on a multidimensional conceptualisation of teacher resilience, this study combined variable-centred and person-centred analyses to explore how resilience is organised within foreign language teachers and how it relates to their emotional and professional adaptation. Four resilience profiles were identified—Frail, Hardcore, Outreaching, and Balanced—of which three formed an ordered resilience gradient and one displayed a crossover configuration of low internal but high relational resources. Profile membership was systematically associated with eleven of twelve adaptive outcomes: more resilient teachers reported more adaptive emotion regulation, more authentic emotional labour, greater subjective wellbeing, and stronger professional identity, whereas surface acting was unrelated to resilience. The findings underscore that teacher resilience matters for foreign language teachers' adaptation not only in degree but also, potentially, in kind.

### 6.2 Theoretical implications

The study carries several theoretical implications. First, it extends the teacher resilience literature by demonstrating the added value of a person-centred lens. Beyond the uniformly positive associations identified in the variable-centred analysis, the latent profile analysis revealed within-sample heterogeneity—most teachers arrayed along a severity gradient, alongside a small configuration in which relational and personal resources dissociated. Second, the results refine the ecological view of teacher resilience by suggesting that internal resources, particularly personal competences and meaning-related strengths, may constitute the component most tightly coupled to adaptive emotional processing and intrinsic professional rewards. By contrast, relational resources may chiefly sustain belonging and behavioural engagement. Third, by situating emotion regulation, emotional labour, wellbeing, and professional identity within a single framework, the study offers an integrated account of how a foundational adaptive resource is linked to both the processes and the outcomes of teachers' adaptation in foreign language education, a domain whose distinctive emotional and intercultural demands have been increasingly recognised (Tao et al., 2024; Turnbull and Arnett, 2002).

### 6.3 Practical implications

The findings also suggest practical directions for schools and teacher educators. The size of the Frail profile—roughly one third of the sample—indicates that a substantial minority of foreign language teachers experience scarce resources across the board and constitute a natural priority for support. As reappraisal most clearly distinguished the profiles, professional development should prioritise helping teachers cognitively reframe classroom and institutional stressors, while also providing mastery-oriented experiences and mentoring to strengthen their personal competences and teaching efficacy. The strong profile differences in school connectedness point to the importance of organisational structures, such as subject-based teaching research groups and supportive leadership, for embedding teachers in professional communities. At the same time, the Outreaching configuration offers a cautionary nuance: the presence of supportive families and peers does not guarantee positive inner functioning. Support initiatives should therefore pair relational support with deliberate competence- and meaning-building, rather than assuming that external support alone suffices. Finally, given the emotional demands specific to language teaching—sustaining target-language interaction, managing learner anxiety, and providing continuous corrective feedback—teacher education programmes in foreign language education may benefit from explicitly addressing emotional labour and equipping teachers with adaptive regulatory strategies. For rural schools, resilience-oriented professional development should not rely merely on individual coping workshops. It should also strengthen school-based communities, mentoring networks, and subject-based teaching research groups, because rural teachers may have fewer opportunities for external professional exchange. Online professional communities and regional teacher learning networks may be particularly useful for rural foreign language teachers, who tend to face limited access to specialist support. Because role overload and job stress have been negatively associated with EFL teacher immunity, resilience-oriented interventions should also address workload and organisational stressors rather than placing responsibility for adaptation solely on individual teachers (Zohrabi and Paydar, 2025).

### 6.4 Limitations and delimitations of the study

Several limitations should be considered during the interpretation of findings. First, the cross-sectional design precludes causal inference. In thise sense, resilience configurations and emotional or professional adaptation may influence one another reciprocally. Second, all constructs were assessed by self-report, which may have introduced common-method and social-desirability bias. Third, the use of convenience sampling and voluntary response means that a response rate could not be calculated and that the sample cannot be claimed to be statistically representative of all rural junior secondary English teachers in Fujian. Fourth, the sample came from one Chinese province and was predominantly female, limiting generalisation to other regions, school sectors, and cultural contexts. Finally, the Outreaching profile contained only 14 teachers, and its estimated means and standard errors were therefore less precise than those of the larger profiles.

The study was deliberately delimited to in-service junior secondary English teachers in Fujian Province, with a substantive focus on teachers working in rural or rural-adjacent schools. It examined teacher resilience through four dimensions of the Teachers' Resilience Scale and focused on selected indicators of emotional and professional adaptation. It did not examine demographic predictors of profile membership, school-level effects, or formal urban-rural contrasts. These choices provided a focused test of the proposed framework but necessarily restricted the breadth and representativeness of the conclusions.

Future studies should replicate the profile solution in larger, multi-site samples, use more precise indicators of rurality and school resources, and incorporate observational, peer-, student-, or administrator-reported data. Longitudinal designs, including latent transition analysis, would help establish profile stability and temporal ordering, while multilevel and urban–rural comparative designs could clarify how school and community conditions shape resilience configurations.

## Data Availability

The raw data supporting the conclusions of this article will be made available by the authors, without undue reservation.

## References

[B1] Arslan O. Çelikdemir K. Haser Ç. (2025). Mathematics-related teacher identity profiles of preservice elementary school teachers and middle school mathematics teachers: a latent class analysis. Teach. Teach. Educ. 165:105159. doi: 10.1016/j.tate.2025.105159

[B2] Asparouhov T. Muthén B. (2014). Auxiliary variables in mixture modeling: three-step approaches using Mplus. Struct. Equ. Model. 21, 329–341. doi: 10.1080/10705511.2014.915181

[B3] Bai B. Li J. (2025). Preschool teachers' professional wellbeing, emotion regulation, and professional identity: a multi-level latent profile approach. Teach. Teach. Educ. 157:104965. doi: 10.1016/j.tate.2025.104965

[B4] Beijaard D. Meijer P. C. Verloop N. (2004). Reconsidering research on teachers' professional identity. Teach. Teach. Educ. 20, 107–128. doi: 10.1016/j.tate.2003.07.001

[B5] Beltman S. Mansfield C. Price A. (2011). Thriving not just surviving: a review of research on teacher resilience. Educ. Res. Rev. 6, 185–207. doi: 10.1016/j.edurev.2011.09.001

[B6] Daniilidou A. Platsidou M. (2018). Teachers' resilience scale: an integrated instrument for assessing protective factors of teachers' resilience. Hell. J. Psychol. 15, 15–39. doi: 10.1037/t70816-000

[B7] Grandey A. A. (2000). Emotion regulation in the workplace: a new way to conceptualize emotional labor. J. Occup. Health Psychol. 5, 95–110. doi: 10.1037/1076-8998.5.1.95 10658889

[B8] Gross J. J. (1998). The emerging field of emotion regulation: an integrative review. Rev. Gen. Psychol. 2, 271–299. doi: 10.1037/1089-2680.2.3.271

[B9] Gross J. J. John O. P. (2003). Individual differences in two emotion regulation processes: implications for affect, relationships, and wellbeing. J. Pers. Soc. Psychol. 85, 348–362. doi: 10.1037/0022-3514.85.2.348 12916575

[B10] Gu Q. Day C. (2007). Teachers resilience: a necessary condition for effectiveness. Teach. Teach. Educ. 23, 1302–1316. doi: 10.1016/j.tate.2006.06.006

[B11] Hascher T. Beltman S. Mansfield C. (2021). Teacher wellbeing and resilience: toward an integrative model. Educ. Res. 63, 416–439. doi: 10.1080/00131881.2021.1980416

[B12] Hascher T. Waber J. (2021). Teacher wellbeing: a systematic review of the research literature from the year 2000–2019. Educ. Res. Rev. 34:100411. doi: 10.1016/j.edurev.2021.100411

[B13] Hochschild A. R. (1983). The Managed Heart: Commercialization of Human Feeling. Berkeley: University of California Press.

[B14] Howard M. C. Hoffman M. E. (2018). Variable-centered, person-centered, and person-specific approaches: where theory meets the method. Organ. Res. Methods 21, 846–876. doi: 10.1177/1094428117744021

[B15] Khalili A. Dobakhti L. Zohrabi M. (2024). Scrutinizing the predicting factors in native and non-native English instructors' teacher immunity. J. Res. Appl. Linguist. 15, 62–74. doi: 10.22055/rals.2023.43835.3061

[B16] Khalili A. Zohrabi M. Dobakhti L. (2025). A cross-cultural scrutiny of predictors of English teachers' immunity in the contexts of Iran, France, and the United States. SAGE Open 15, 1–18. doi: 10.1177/21582440251367151

[B17] Kohler M. (2015). Teachers as Mediators in the Foreign Language Classroom. Bristol, England: Multilingual Matters. doi: 10.21832/9781783093076

[B18] Lei J. Ye W. (2025). Exploring Chinese university students' motivation and engagement in English medium instruction: a latent profiling approach. System 131:103676. doi: 10.1016/j.system.2025.103676

[B19] Li J. B. Xu Y. Sun J. Qiu S. Zhang R. Yang A. (2025). A multilevel latent profile analysis to job demands, job resources, and personal resources in early childhood educators: implications for multidimensional wellbeing. J. Sch. Psychol. 109:101405. doi: 10.1016/j.jsp.2024.101405 40180459

[B20] Liddicoat A. Scarino A. (2013). Intercultural Language Teaching and Learning. Malden, MA: Wiley-Blackwell. doi: 10.1002/9781118482070

[B21] Lin Y. Liu W. (2025). Beginning teachers' innovative work behavior and stress toward change: examining the roles of adaptability and contextual influences. Teach. Teach. Educ. 165:105164. doi: 10.1016/j.tate.2025.105164

[B22] Liu H. Yao Y. (2026). A latent profile analysis of academic emotions: associations with perceived classroom affordance and engagement among Chinese ethnic minority EFL students. Eur. J. Educ. 61:e70632. doi: 10.1111/ejed.70632

[B23] Long Z. Zhu R. (2025). A latent profile analysis of emotional intelligence and its relationship with L2 student writing feedback literacy. System 133:103773. doi: 10.1016/j.system.2025.103773

[B24] Mansfield C. F. Beltman S. Broadley T. Weatherby-Fell N. (2016). Building resilience in teacher education: an evidenced informed framework. Teach. Teach. Educ. 54, 77–87. doi: 10.1016/j.tate.2015.11.016

[B25] Marsh H. W. Lüdtke O. Trautwein U. Morin A. J. S. (2009). Classical latent profile analysis of academic self-concept dimensions: synergy of person- and variable-centered approaches to theoretical models of self-concept. Struct. Equ. Model. 16, 191–225. doi: 10.1080/10705510902751010

[B26] Morin A. J. S. Bujacz A. Gagné M. (2018). Person-centered methodologies in the organizational sciences: introduction to the feature topic. Organ. Res. Methods 21, 803–813. doi: 10.1177/1094428118773856

[B27] Nylund K. L. Asparouhov T. Muthén B. O. (2007). Deciding on the number of classes in latent class analysis and growth mixture modeling: a monte carlo simulation study. Struct. Equ. Model. 14, 535–569. doi: 10.1080/10705510701575396

[B28] Nylund-Gibson K. Choi A. Y. (2018). Ten frequently asked questions about latent class analysis. Transl. Issues Psychol. Sci. 4, 440–461. doi: 10.1037/tps0000176

[B29] Pan Z. Wang Y. (2025). From technology-challenged teachers to empowered digitalized citizens: exploring the profiles and antecedents of teacher AI literacy in the Chinese EFL context. Eur. J. Educ. 60:e70020. doi: 10.1111/ejed.70020

[B30] Pearce J. Morrison C. (2011). Teacher identity and early career resilience: exploring the links. Aust. J. Teach. Educ. 36, 48–59. doi: 10.14221/ajte.2011v36n1.4

[B31] Renshaw T. L. Long A. C. J. Cook C. R. (2015). Assessing teachers' positive psychological functioning at work: development and validation of the teacher subjective wellbeing questionnaire. Sch. Psychol. Q. 30, 289–306. doi: 10.1037/spq0000112 25642703

[B32] Sinha P. Calfee C. S. Delucchi K. L. (2021). Practitioner's guide to latent class analysis: methodological considerations and common pitfalls. Crit. Care Med. 49, 63–79. doi: 10.1097/CCM.0000000000004710 PMC7746621 33165028

[B33] Spurk D. Hirschi A. Wang M. Valero D. Kauffeld S. (2020). Latent profile analysis: a review and “how to” guide of its application within vocational behavior research. J. Vocat. Behav. 120:103445. doi: 10.1016/j.jvb.2020.103445

[B34] Sun Y. Yin H. (2025). Profiles of teacher self-efficacy and their relations to teacher demographics and affective wellbeing: a social cognitive perspective. Teach. Teach. Educ. 154:104855. doi: 10.1016/j.tate.2024.104855

[B35] Tao J. Xu Y. Gao X. A. (2024). Teacher emotions and agency enactment in online teaching. Teach. Teach. Educ. 137:104389. doi: 10.1016/j.tate.2023.104389

[B36] Turnbull M. Arnett K. (2002). Teachers' uses of the target and first languages in second and foreign language classrooms. Annu. Rev. Appl. Linguist. 22, 204–218. doi: 10.1017/S0267190502000119

[B37] Yin H. (2012). Adaptation and validation of the teacher emotional labour strategy scale in China. Educ. Psychol. 32, 451–465. doi: 10.1080/01443410.2012.674488

[B38] Yin H. (2015). The effect of teachers' emotional labour on teaching satisfaction: moderation of emotional intelligence. Teach. Teach. 21, 789–810. doi: 10.1080/13540602.2014.995482

[B39] Yin H. Huang S. Chen G. (2019). The relationships between teachers' emotional labor and their burnout and satisfaction: a meta-analytic review. Educ. Res. Rev. 28:100283. doi: 10.1016/j.edurev.2019.100283

[B40] Zhou S. Slemp G. R. Vella-Brodrick D. A. (2025). Profiling pre-service teachers' motivational and behavioral characteristics: relationships with psychological wellbeing and occupational commitment. Teach. Teach. Educ. 164:105041. doi: 10.1016/j.tate.2025.105041

[B41] Zohrabi M. Khalili A. (2024). The philosophy of teacher immunity: EFL teachers' perspectives. J. Philos. Investig. 17, 330–346. doi: 10.22034/JPIUT.2024.59889.3664

[B42] Zohrabi M. Maleki M. (2025). Emotional intelligence, motivation and job satisfaction among EFL English practitioners. Lang. Identity Digit. Real. 1, 29–49. doi: 10.22034/lidr.2025.2067568.1015

[B43] Zohrabi M. Paydar Z. (2025). Iranian EFL practitioners' issues at private institutes and state schools. Teach. Engl. Lang. 19, 81–118.

